# Depth Estimation and Semantic Segmentation from a Single RGB Image Using a Hybrid Convolutional Neural Network

**DOI:** 10.3390/s19081795

**Published:** 2019-04-15

**Authors:** Xiao Lin, Dalila Sánchez-Escobedo, Josep R. Casas, Montse Pardàs

**Affiliations:** 1 Visual Interactions and Communication Technologies (Vicomtech), 20009 Donostia/San Sebastián, Spain; 2 Image Processing Group, TSC Department, Technical University of Catalonia (UPC), 08034 Barcelona, Spain; dalila.s2510@gmail.com (D.S.-E.); josep.ramon.casas@upc.edu (J.R.C.); montse.pardas@upc.edu (M.P.)

**Keywords:** depth estimation, semantic segmentation, convolutional neural networks, hybrid architecture

## Abstract

Semantic segmentation and depth estimation are two important tasks in computer vision, and many methods have been developed to tackle them. Commonly these two tasks are addressed independently, but recently the idea of merging these two problems into a sole framework has been studied under the assumption that integrating two highly correlated tasks may benefit each other to improve the estimation accuracy. In this paper, depth estimation and semantic segmentation are jointly addressed using a single RGB input image under a unified convolutional neural network. We analyze two different architectures to evaluate which features are more relevant when shared by the two tasks and which features should be kept separated to achieve a mutual improvement. Likewise, our approaches are evaluated under two different scenarios designed to review our results versus single-task and multi-task methods. Qualitative and quantitative experiments demonstrate that the performance of our methodology outperforms the state of the art on single-task approaches, while obtaining competitive results compared with other multi-task methods.

## 1. Introduction

Semantic segmentation and depth information are intrinsically related, and both pieces of information need to be considered in an integrated manner to succeed in challenging applications, such as robotics [1] or autonomous navigation [2]. In robotics, performing tasks in interactive environments requires identification of objects as well as their distance from the camera. Likewise, autonomous navigation applications need a 3D reconstruction of the scene as well as semantic information to ensure that the agent device has enough information available to carry out the navigation in a safe and independent manner. Although RGB-D sensors are currently being used in many applications, most systems only provide RGB information. Therefore, addressing depth estimation and semantic segmentation under a unified framework is of special interest.

On the other hand, deep learning techniques have shown extraordinary success in both tasks [3] in recent years. In this context, the feature-extraction process for a specific task is modeled as a parameter estimation problem in Convolutional Neural Networks (CNNs) which is based on a set of training data. In other words, the feature extractors are created by learning from the prior knowledge that we have. This provides a possibility of combining different tasks (different sources of prior knowledge) when training the feature extractors, in particular for highly correlated tasks such as depth estimation and semantic segmentation. Specifically, the idea of integrating the depth estimation and semantic segmentation into a sole structure is motivated by the fact that both segmentation information and depth maps represent geometrical information of a scene. In this manner, the feature extractors can be better trained due to the enriched prior knowledge.

In this paper, we introduce a hybrid convolutional network that integrates depth estimation and semantic segmentation into a unified framework. We propose to build a model where the features extracted are suitable for both tasks, thus leading to an improved accuracy in the estimated information. One of the main advantages of the proposed approach is the straightforward manner semantic segmentation and depth map are estimated from a single image, providing a feasible solution to these problems.

## 2. Related Work

Depth estimation and semantic segmentation are two widely studied problems in the image processing community and recently have been tackled through deep learning techniques due to its successful results in terms of accuracy and efficiency. This section makes a review of the state of the art introducing, first, single-task approaches, and, afterwards, methods focused on solving multiple tasks.

### 2.1. Single-Task Approaches

#### 2.1.1. Semantic Segmentation

Semantic segmentation is a methodology which approaches the image segmentation problem by performing pixel-level classifications. Compared to the traditional image segmentation approaches, such as superpixel segmentation methods [4,5], active contour methods [6,7] and watershed segmentation methods [8,9], it introduces semantics in an image segmentation process by employing a classifier trained on the annotated data. Although semantic segmentation methods generally have limited genericity due to the predefined semantics in the annotations, the advantage of semantic segmentation is obvious. The introduced semantics provide higher level knowledge which helps obtain more meaningful segments in comparison to homogeneous regions.

Before CNN-based techniques were applied to semantic segmentation; handcrafted features were usually employed to represent pixels when training the classifier [10]. The emergence of CNN-based techniques provide an approach that trains neural networks to extract features with higher discriminative power. One of the first well known works that applies CNNs to semantic segmentation is Fully Convolutional Networks (FCN) [11]. It popularizes CNN architectures for dense predictions without any fully connected layers. This allowed segmentation maps to be generated for images of any size and it also reduces the number of parameters in the architecture since no fully connected layers are involved. Almost all the subsequent state-of-the-art approaches on semantic segmentation adopted this paradigm.

Apart from fully connected layers, one of the main problems of using CNNs for segmentation are the pooling layers. Pooling layers increase the field of view and are able to aggregate the context while discarding the ‘where’ information. However, semantic segmentation requires the exact alignment of class maps and thus, needs the ‘where’ information to be preserved. Two different classes of architectures evolved in the literature to tackle this issue.

The first one is the encoder-decoder architecture. The encoder gradually reduces the spatial dimension with pooling layers and the decoder gradually recovers the object details and spatial dimension. There are usually shortcut connections from the encoder to the decoder to help the decoder recover the object details better. U-Net [12] is a popular architecture from this class. It consists of a contracting path to capture context in the encoder and a symmetric expanding path from the encoder layers to the decoder layers that enables precise localization. Seg-Net [13] is proposed based on FCN. It introduces more shortcut connections between the encoder and the decoder. Furthermore, it copies the indices from the max-pooling layers in the encoder to the decoder instead of copying the encoder features as in FCN, which makes easier for SegNet to recover the spatial information and provides more memory efficiency than FCN. Ghiasi et al. [14] present a Laplacian pyramid for semantic segmentation refinement incorporating, into the decoding step, the spatial information contained in the high-resolution feature maps to keep the spatial information destroyed after pooling. Thus, a better dense pixel-accurate labeling is obtained.

Architectures in the second class use what are known as dilated/atrous convolutions [15,16,17]. Pooling layers help in classification networks because they increase the receptive field of a network. However, as mentioned, this is not suitable for a semantic segmentation task since pooling drops the spatial information and decreases the resolution. Dilated/Atrous convolutions can compute responses at all image positions with an *n* times larger receptive field if the full resolution image is convolved with a filter ‘with holes’, in which the original filter is upsampled by a factor *n*, and zeros are introduced in between filter values. Although the effective filter size increases, it is only necessary to take into account the non-zero filter values, hence both the number of filter parameters and the number of operations per position stay constant.

#### 2.1.2. Depth Estimation

One of the first works to tackle the depth estimation problem using CNNs is the one presented in [18]. They used a novel network architecture made of two main components. First, a coarse-scale network estimates a low-resolution depth map from a single image. Then, this depth estimation along with the original image becomes the input of the fine-scale network. In this way, the local network can incorporate finer-scale details in the global prediction. Additionally, they also apply a scale-invariant error to help measure depth relations instead of scale.

Likewise, a similar work based on [18] is presented in [19]. In this approach the authors included an extra part to the model presented by [18] that estimates gradient information. The idea behind this additional part is to improve the fine-tuning part by adding gradient information along with the global depth estimation and the input image. Additionally, a normalized loss function was applied resulting in a better depth estimation.

### 2.2. Multi-Task Approaches

Approaches addressing depth estimation and semantic segmentation with multi-task learning schemes are receiving large attention due to its potential of improving the performance of multiple tasks. The idea of merging tasks in one architecture is motivated by the fact that different correlated tasks commonly share some basic attributes in the parsing process. Approaching them together may be mutually beneficial. In practice, multi-task approaches in the state of the art seek to extract features suitable to perform diverse tasks at a time, which lead to an improvement in both estimated information and simplification of systems where multiple modalities are required, such as autonomous navigation [2], robotics [1] or augmented reality [20].

In [21], the authors provide a common network which can be used for different tasks, including the estimation of depth map, surface normals, and semantic segmentation. Although these tasks are not all addressed jointly, it proves that a network for a specific task can be obtained by fine-tuning a network with the same architecture trained for another correlated task. The results obtained by [21] outperformed the ones presented in [18] proving how the strategy of tackling multiple tasks with a common network may lead to a better performance.

In [22] a unified framework was proposed, which incorporates global and local prediction where the consistency between depth and semantic segmentation is learned through a joint training process. From an input image, they first used a CNN to jointly predict a global depth map and semantic labels. Then, they decompose the image into local regions to train another CNN which predicts the depth map and the semantic labels for each region. With global and local predictions, they re-formulate the problem into a two-layer hierarchical conditional random field to produce the final depth and semantic map.

A more recent multi-task approach is introduced in [23]. The methodology proposed in this work makes initial estimations for depth and semantic label at a pixel level through a joint network. Later, depth estimation is used to solve possible confusions between similar semantic categories and thus to obtain the final semantic segmentation.

Another multi-task approach by Teichmann et al. [24] presents a network architecture named MultiNet that can perform classification, semantic segmentation, and detection simultaneously. They incorporate these three tasks into a unified encoder-decoder network where the encoder stage is shared among all tasks and specific decoders for each task produce outputs in real time. These work efforts were focused on improving the computational efficiency for real-time applications as autonomous driving.

A similar approach is Pixel-Level Encoding and Depth Layering (PLEDL) [25], this work extended a FCN [11] with three output channels jointly trained to estimate semantic labeling, direction to the instance center and depth at pixel level.

Table 1 presents a brief comparison on the pros and cons between different types of methods. Traditional image segmentation approaches [4,5,6,7,8,9] usually perform low-level segmentation, which obtain the segments with more general assumptions, such as local homogeneity. On the other hand, semantic segmentation methods [10,11,13,16,26] improve image segmentation by introducing semantic annotations, which provide higher level meaning (semantics at object level) rather than low-level features exploited in traditional methods. Approaches under multi-task learning schemes, such as the proposed approach and [21,25] exploit the correlation between semantic segmentation and depth estimation to benefit each of the tasks, which generate both image segmentation and depth estimation taking as input a single-color image. Unlike the multi-task methods in the state of the art [21,25], the proposed approach focuses on separating the commons and distinctions between the two tasks, which obtains promising results shown in our experiments.

### 2.3. Our Proposal

Multi-task approaches aim to directly estimate the segmentation and depth maps from an input color image by unifying CNNs working for a single task into a sole hybrid convolutional neural network. Most of the state-of-the-art works unify tasks under a feature-extraction block whose output becomes the input of a group of decoders designed to carry out each task.

In our preliminary work [27], we presented a hybrid network for a multi-task learning scheme that benefits both semantic segmentation and depth estimation, and its application to autonomous driving scenes. This hybrid network employs a global depth estimation network to estimate separately the global layout of a scene from the input image additionally to the common feature extraction.

**Figure 1:**
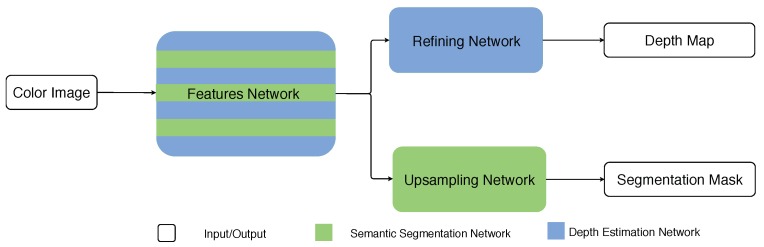
Architecture 1.

**Figure 2:**
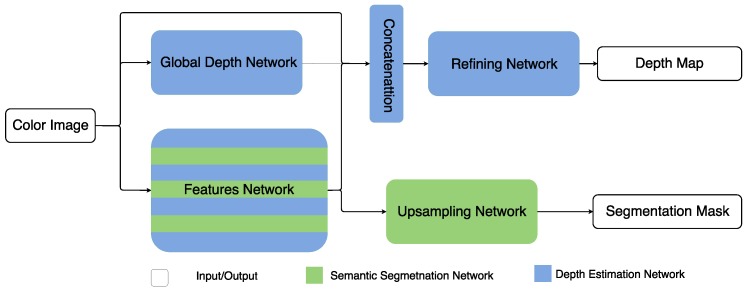
Architecture 2.

In this paper, we focus on comparing different hybrid network unifying strategies and investigating how those two tasks help each other. More specifically, we employ two unifying strategies, one from the hybrid architecture proposed in our previous work [27] and the other from the state-of-the-art works [21,23,24]. In the experiments, we compare the performances obtained from different hybrid architectures, named HybridNet A1 and A2 (see Figure 1 and Figure 2), by applying different unifying strategies to the same single-task architectures, in order to clarify how the two tasks help each other in a hybrid system. We also apply them to the more challenging indoor scenes to verify the validity of the unifying strategy.

The rest of the paper is organized as follows: in Section 3 we introduce the proposed methodology; the detailed explanation of the proposed architectures is presented in Section 4, as well as the training details. In Section 5, we present the experiment results of our approach in different datasets and compare our approach with state-of-the-art approaches. Finally, conclusions are drawn in Section 6.

## 3. Hybrid Convolutional Framework

In this section, a general explanation of our hybrid convolutional neural network and its application to depth estimation and semantic segmentation is presented. To this end, a description about the two single-task architectures [16,19] employed in our approach is first presented. Then, we describe the proposed hybrid architecture along with a discussion to approach the problem of how to unify two tasks under one sole framework.

**Figure 3:**
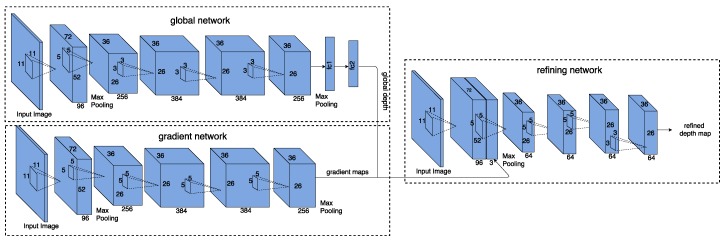
Depth estimation network.

The depth estimation architecture [19], denoted as DepthNet in this paper, is made of three components: global depth network, gradient network, and refining network, as shown in Figure 3. These three components all follow AlexNet structure. DepthNet first estimates a depth map of the scene at a global level from the single input RGB image via a global depth network. Meanwhile, it predicts two depth gradient maps from the input RGB image via a gradient network. Finally, a refining network uses the input image along with depth gradient maps to locally refine the global depth map and thus produce a better detailed depth map. As explained in [19], the three components in DepthNet are trained separately. For training the global depth network, the downsampled depth maps are used as the ground truth. Beside the global depth network, the gradient network is trained based on the magnitude of depth gradient on *x* and *y* direction computed from the depth map. Along with the global depth network and gradient network, the refining network is again trained on the downsampled depth maps in the training data.

There are two main reasons to consider employing DepthNet as the depth estimation component in our approach: (1) DepthNet follows the state-of-the-art framework for depth estimation which is representative for a bunch of methods. (2) DepthNet has a modularized architecture, which allows us to analyze each of the components in it and better integrate DepthNet into a hybrid architecture.

**Figure 4:**
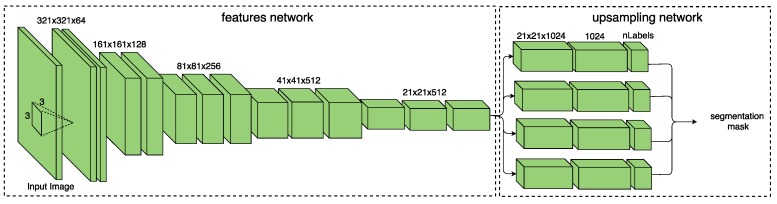
Semantic segmentation network.

The semantic segmentation architecture [16] shown in Figure 4, is divided into two main parts: feature network and atrous upsampling network. For the feature network, it follows the VGGNet architecture proposed in [28]. It is in charge of extracting robust features from the input image, which benefits from the deep structure of the network. On the other hand, the atrous upsampling network is a group of atrous spatial pyramid pooling layers [16] which outputs a class score map with the number of channels equal to the number of labels. Atrous upsampling layers allows us to explicitly control the resolution at which feature responses are computed within the architecture, while enlarging the field of view of filters to incorporate larger context in the semantic segmentation task. The semantic segmentation architecture is denoted as DeepLab-Atrous Spatial Pyramid Pooling (DeepLab-ASPP) in this paper. In DeepLab-ASPP, all parts are trained together.

DeepLab-ASPP is employed as the semantic segmentation component in our approach due to its outstanding performance in this task.

### Unifying Single-Task Architectures for Multi-Tasks

Considering the functionality of each component in DepthNet and DeepLab-ASPP, we propose and compare two different hybrid architectures for the joint depth estimation and semantic segmentation task.

*Architecture 1*: An intuitive way to unify two tasks in a sole architecture is to totally share the feature-extraction process for both tasks. It follows the idea from the most representative architectures in the state of the art [21,23,24], in which a common convolutional network is shared for extracting features. Following the feature-extraction block, customized layers are used for each task, to decode the commonly extracted features and apply them in different tasks. Sharing the feature-extraction process for different tasks with a common convolutional network links the two tasks, since the parameters of the shared network are optimized with respect to the losses defined on both tasks in the training phase. The advantage of this architecture is obvious. Since most of the layers are shared for both tasks, less parameters are involved in the training process, which makes it easy to be trained. In practice, we exploit the VGG structure [28] as the feature-extraction network for both tasks. Based on the extracted features, the atrous upsampling network in DeepLab-ASPP is employed for the semantic segmentation task, while the refining network in DepthNet is leveraged as the decoder for the depth estimation task. We denote the architecture 1 as HybridNet A1 in the rest of this paper.

*Architecture 2*: The motivation of building this architecture is to further clarify the common and specific attributes in the two tasks. Thus, we build the hybrid network by substituting the gradient network in DepthNet by a common feature-extraction network for the two tasks, while keeping the global depth estimation network only for the depth task. The advantages of this hybrid architecture are two-fold. On one hand, the strong power of extracting object information from a color image learned in the semantic segmentation task can also benefit depth layering when predicting a depth map, while the strong power of extracting rich depth boundaries from a color image learned in the depth estimation task is shared in the semantic segmentation task to improve segmentation accuracy on object boundaries. On the other hand, the global layout of the scene which is more relevant in depth estimation than in semantic segmentation is estimated independently by a global network in the depth estimation task. This avoids interfering the common feature extraction for both tasks. In practice, we keep the global network and refining network in DepthNet without changes, while replacing the structure of the gradient network with the VGG structure, in order to keep the structure consistent with the feature network in DeepLab-ASPP. We denote the architecture 2 as HybridNet A2 in the rest of this paper.

## 4. Architecture Details

Since the proposed architectures are assembled with basic components in the two single-task architectures, we explain the detail of the proposed architectures by describing the two single-task architectures in this section.

### 4.1. Depth Estimation Network

As described in Section 3, the depth estimation network is modularized to calculate a refined depth map from a single input image through a three stages convolutional network. As shown in Figure 3, the global depth network is formed by 5 convolutional layers and two fully connected layers, which corresponds to the architecture of AlexNet [29]. Following each convolutional layer, a Rectified Learner Unit (ReLU) is introduced as an activation function to provide non-linearity to the system. Local normalization is also performed after each convolutional layer in a Local Response Normalization layer (LRN), which helps the generalization of the system. Max-pooling layers are placed after the first and the last convolutional layer to provide basic translation invariance to the internal representation and reduce the number of parameters of the system. In this network max-pooling is performed over a 3×3 window with a stride of 2. Since the global depth network aims at describing the global layout of the scene, we introduce two fully connected layers following the last convolutional layer, to capture the information contained in the intermediate representation with the full receptive field. In practice, 1024 neurons are included in the first fully connected layer while 1681 neurons are included in the second fully connected layer. We reshape the 1681 neurons to a 41 by 41 matrix which is treated as the output of the global depth network. In this manner, we predict a global depth map with $\frac{1}{8}$ resolution for an input image with size 321 by 321.

The gradient network aims at estimating depth gradient from an input color image. In practice, we employ the same architecture used in global depth estimation except for the fully connected layers.

Finally, the refining network takes the concatenation of the output from the global depth network, the gradient network, and the color image as input and computes a refined depth map. The refining network improves the rough estimate from the global depth network, using gradients estimated by the gradient network and an input color image. In practice, the first convolutional layer processes the input color image, followed by a ReLU layer, an LRN layer, and a max-pooling layer, which produces the feature maps extracted from the color image. These feature maps are concatenated with outputs of the global depth network and the gradient network, then are fed to the remaining four convolutional layers. Each of them is followed by a ReLU layer. The output from the 5th convolutional layer in the refining network is treated as the output (a refined depth map with size 81 by 81).

### 4.2. Semantic Segmentation Network

Figure 4 presents an overview of semantic segmentation network. This figure shows in a detailed manner how the input image is processed by first going through a group of convolutional layers for feature extraction (feature network) and then through an upsampling procedure which finally provides the segmentation map (upsampling network). Dividing this architecture into two parts helps us to understand it as a single-task network but also how it can be integrated into a hybrid model.

The feature network contains 5 groups of convolutional layers, forming a deep architecture. All these convolutional layers have the same kernel size 3×3. For simplicity, we only plot the convolutional kernel in the first convolutional layer in the feature network. Following each convolutional layer, a ReLU layer is provided as the activation function. Pooling layers are placed after each group of convolutional layers to reduce the computational cost by downsampling the internal representation, as well as to provide basic translation invariance to the internal representation.

On the other hand, the atrous upsampling network contains 4 parallel groups of three convolutional layers, to perform upsampling operation at different scales. Each branch upsamples the output from the feature network at the first convolutional layer with an atrous convolution. An atrous convolution employs a dilated convolution template, in which a convolution template is enlarged by filling zeros with respect to a defined rate. In this manner, we can explicitly control the resolution of the upsampling operation and enlarge the field of view of filters to incorporate larger context in the semantic segmentation task without introducing more parameters. In practice, we employ atrous convolutions with rates 6,12,18,24 respectively for each branch. The other 2 convolutional layers in each branch perform 1×1 convolutions, which increases the non-linearity of the decision function without affecting the receptive fields of the convolutional layers. Taking the output of the 4 branches of upsampling layers as input, a soft-max layer produces the final semantic segmentation mask.

### 4.3. Training Details

As explained in the Section 3, the two proposed architectures (HybridNet A1 and A2) are based on DeepLab-ASPP [16] and DepthNet [19]. Although HybridNet A1 and A2 are constructed by merging single-task architectures, the training process for the hybrid architectures are not always performed as in those single-task architectures.

In HybridNet A1, we initialize the feature network and the atrous upsampling network with the model provided by DeepLab [16] which was pre-trained for classification purpose on ImageNet. The other parts in HybridNet A1 are initialized using a Random Number Generator (RNG). The RNG is set to be a Gaussian distribution with zero mean and 0.1 variance.

In HybridNet A2, we initialize the feature network and upsampling network before the training process using again the model provided by DeepLab [16]. Additionally, we initialize the global depth network using the model provided in [19]. The other parts in HybridNet A2 are randomly initialized using the same RNG.

Once we have the initialization for our hybrid architecture, all of its components are trained simultaneously. Both hybrid architectures are trained for 100 K iterations with a learning rate $2.5\times 10^{-6}$, polynomial learning rate decay policy. The momentum is set to 0.9 and weight decay 0.005. The input image is randomly cropped with a size 320×320. We set batch size to 7, regarding the maximum allowance of memory.

The loss function used in both architectures is the same. For the semantic segmentation task, $L_{S}$ is the sum of the cross-entropy terms for each spatial position in the output class score map, being our targets the ground truth labels. All positions and labels of the output class score map are equally weighted in the overall loss function except for those unlabeled pixels which are ignored. The loss function used for the depth estimation task is made by two Euclidean loss layers $L_{D_{abs}}$ and $L_{D_{mvn}}$. $L_{D_{abs}}$ computes the Euclidean distance between absolute values of a depth map in the ground truth and the estimated depth map, while the $L_{D_{mvn}}$ computes the Euclidean distance between estimation and ground truth after performing a mean variance normalization on both of them. $L_{D_{abs}}$ stands for a pixel-level metric which evaluates locally how well the estimated depth value matches the ground truth regardless of the geometry of the scene. On the other hand, $L_{D_{mvn}}$ introduces a global regularization in the depth estimation by aligning depth values in both the estimation and the ground truth to zero mean and unit variance.

The hybrid loss function $L_{H}$ is therefore defined as the linear combination of them:

(1) $$\begin{array}{ccc} L_{H} & = & \alpha L_{S}+\left(L_{D_{abs}}+L_{D_{mvn}}\right) \end{array}$$

where α is the term used to balance the loss functions of the depth estimation and semantic segmentation tasks. In our experiments, α is set to 1000, given an analysis on the values of $L_{D_{abs}}+L_{D_{mvn}}$ and $L_{S}$ respectively, when training them separately in the single-task architectures.

## 5. Experiments

We quantify the performance of the proposed architectures on both semantic segmentation and depth estimation in different scenes using our Caffe implementation. We first evaluate the proposed architectures in road scenes which is of current practical interest for various autonomous driving related problems. Secondly, the proposed architectures are evaluated in indoor scenes which is of immediate interest to possible augmented reality (AR) applications.

### 5.1. Road Scene

In this section, we present the evaluation of the proposed architectures in road scenes. Several road scene datasets are available for semantic parsing [30,31,32]. Since we evaluate the proposed architecture from both semantic segmentation and depth estimation perspective, we employ Cityscapes dataset [32] in our experiment, which provides not only the ground truth of semantic labels but the depth information of each frame. Cityscapes contains 5000 RGB images manually selected from 27 different cities for dense pixel-level annotation to ensure high diversity of foreground objects, background, and overall scene layout. Along with each of the 5000 RGB images, Cityscapes dataset provides the depth map obtained from a stereo vision system. The 5000 images in the dataset are split into 2975 training RGB images of size 1024×2048 along with their corresponding 2D ground truth object labels for 19 outdoor scenes classes and depth information, 500 RGB images for test validation with their corresponding annotations and, for benchmarking purposes, 1525 test RGB images.

In practice, the training process of our approach was performed using the 2975 images of Cityscapes training set that provides a depth map and object labels of 19 classes for each RGB image. To evaluate the performance of the proposed architectures, we group the 500 images of the validation set and the 1525 images of the test set in the Cityscapes dataset into a single evaluation set of 2025 images. In the training phase, images in the training set are shuffled and randomly cropped to fit the input image size of the hybrid architecture. Training data augmentation is done by flipping and mirroring the original images, to enlarge the training set. In the testing phase, we crop the test image with the original size of 1024×2048 into a group of images with the size of 321 by 321 which cover the whole test image while having the minimum overlapped area. These images are tested one by one and grouped to obtain the final prediction of the segmentation mask and depth map. Please note that a score map is obtained for each image, which shows the degree that a pixel belongs to a label. For the overlapped area, we compare the normalized score maps and take the label with higher score as predicted labels on the segmentation mask. Likewise, for the overlapped area, the predicted depth values on the depth map are computed as the mean values.

Our first aim is to determine if the features obtained in the shared part of the proposed architectures solving the two tasks simultaneously provide better results than the ones that we would obtain using two identical networks trained separately. This is why, in addition to the results of the proposed architectures, we present the results obtained by the models that solve these two tasks separately for comparison. The models used to train semantic segmentation and depth estimation independently are denoted as DeepLab-ASPP [16] and DepthNet [19], respectively. We trained these two models using the code provided by the authors with the same training data in Cityscapes dataset and the same training configuration than the proposed architectures. Apart from that, we also compare different ways of unifying single-task architectures proposed in Section 3, to justify whether the unifying strategy is better. Besides, the comparison between the proposed architectures and a hybrid method in the state of the art [25] is also made in Cityscapes dataset. The hybrid approach proposed in [25] is similar to HybridNet A1, in which the encoder network in FCN [11] is employed as the feature network shared by three different tasks and the decoder network in FCN is then employed for each task to decode the commonly extracted features. The three tasks that [25] tackles are semantic segmentation, depth layering, boundary detection, which is similar to our target. However, in the depth layering task, ref. [25] focuses on estimating a depth label for each object, instead of estimating the real depth value of the whole scene at pixel level. This is also the reason that we only compare the performance between our approach and [25] in semantic segmentation. We present the results in our experiments in the following two subsections specifying the evaluation in semantic segmentation and depth estimation, respectively.

#### 5.1.1. Semantic Segmentation

**Figure 5:**
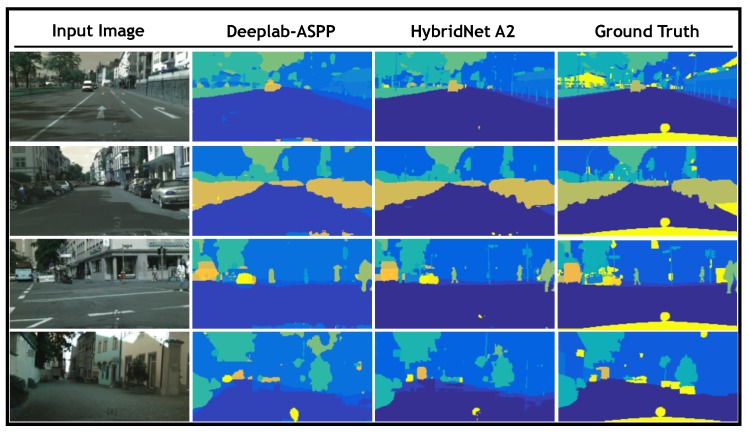
Semantic segmentation qualitative results. A comparison between semantic segmentation estimation against ground truth is presented. From left to right, input image is depicted in the first column. In column 2 the segmentation map estimated by DeepLab-ASPP semantic segmentation network [28] is presented, in column 3 the estimated segmentation map by our hybrid method are presented and finally the ground truth is depicted in column 4.

Figure 5 provides four examples from the evaluation set for visual comparison between the results obtained by our hybrid model and ground truth as well as those obtained by DeepLab-ASPP. The purpose of this figure is to depict the differences between a single-task and a multi-task approach. In Figure 5 the input image is displayed in the first column, second and third columns show the results obtained by DeepLab-ASPP and our hybrid model, respectively. Finally, in the fourth column the ground truth is presented for reference. This figure shows how the segmentation performed by the proposed HybridNet A2 retains with a greater detail the geometrical characteristics of the objects contained in the scene. For instance, in the 3rd row where the shapes of a pedestrian and a car can be better distinguished in the estimation obtained by Hybrid A2 than the one obtained by DeepLab-ASPP.

In addition to qualitative results, we employ three commonly used metrics, to measure quantitatively the segmentation performance: the global accuracy (G), the class average accuracy (C) and mean intersection over union (mIoU). The global accuracy counts the percentage of pixels which are correctly labeled with respect to the ground truth labeling. The class average accuracy is the mean of the pixel accuracy in each class. The mean intersection over union measures the average Jaccard scores over all classes. Table 2 presents the quantitative results and confirms that the proposed HybridNet outperforms the results obtained by DeepLab-ASPP. Please note that the global accuracy and the class average accuracy evaluation of PLEDL are not provided due to the unavailability of the source code, whereas the evaluation of mIoU is reported in [25].

The improvements obtained by our method against DeepLab-ASPP confirm the hypothesis that sharing the feature-extraction network between tasks leads to an improvement in terms of segmentation accuracy. The strategy of unifying two single-task architectures affects the segmentation performance of hybrid methods. HybridNet A2 where common and specific attributes between two different tasks are better clarified outperforms HybridNet A1 in which the feature-extraction process is totally shared for the two tasks. The improvement that HybridNet A1 obtains against DeepLab-ASPP is very limited (HybridNet A1 58.1% mIoU against DeepLab-ASPP 58.02% mIoU); however, Hybrid A2 improves the mIoU by around 8%. We also compare our architectures against a state-of-the-art hybrid method [25] in Table 2. HybridNet A2 has a better segmentation performance in all three metrics, than the work in [25]. For additional evaluation, comparisons between our approach against other well adopted single-task methods [11,13,16,33] are presented in Table 2.

#### 5.1.2. Depth Estimation

**Figure 6:**
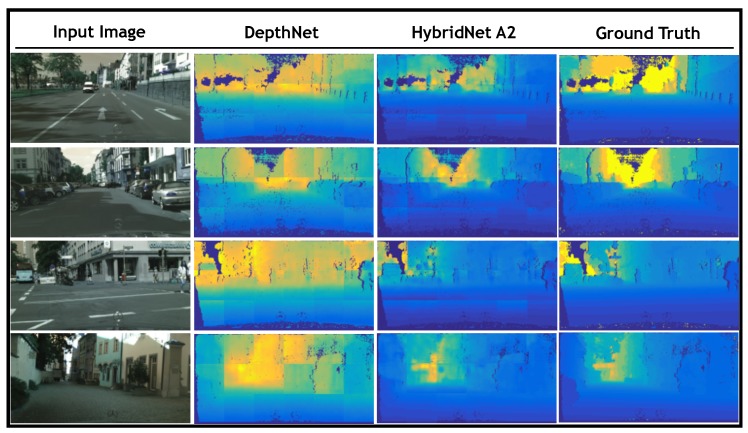
Depth estimation qualitative results. A visual comparison between the estimated depth maps against the ground truth is presented. In the first column the input image is presented, columns 2 and 3 depict the estimated depth maps obtained by DepthNet in [19] and our hybrid model A2, respectively. Finally, ground truth is presented in column 4.

For depth estimation evaluation, Figure 6 presents a visual comparison of the results obtained by Hybrid A2 as well as those obtained by the single-task approach presented in [19] against the ground truth. The figure displays, row-wise the same four examples depicted in Figure 5. Figure 6 depicts the input image in the first column, the depth map obtained by DepthNet in the second column, while third and fourth columns show the depth map obtained by HybridNet A2 and ground truth, respectively. Note how the results obtained by Hybrid A2 are more consistent with the ground truth than those obtained by DepthNet in terms of the depth layering.

Additionally to qualitative analysis, we evaluate the performance of our methodology for depth estimation employing 6 commonly used metrics: Percentage of Pixel (PP), Mean Variance Normalized Percentage of Pixel (PP-MVN), Absolute Relative Difference (ARD), Square Relative Difference (SRD), Linear Root Mean Square Error (RMSE-linear), Log Root Mean Square Error (RMSE-log) and Scale-Invariant Error (SIE).

Table 3 shows the definition for these metrics employed in the evaluation process. *d* and $d^{*}$ represent the estimated depth and ground truth, respectively. *N* stands for the number of pixels with valid depth value in the ground truth depth map.

In the quantitative experiment, we compare the proposed hybrid architectures and DepthNet. Table 4 shows the quantitative results of the proposed hybrid architectures and DepthNet under the different evaluation metrics introduced above. HybridNet A2 outperforms in 6 out of 9 metrics, which proves that training the feature-extraction network for the simultaneous tasks of semantic segmentation and depth estimation also improves the depth estimation results. The better performance of HybridNet A2 in comparison to DepthNet illustrates that the shared features obtained with the semantic segmentation task in HybridNet A2 have richer information and are more relevant in the depth estimation task than the information extracted from the depth gradient in DepthNet. The comparison between Hybrid A2 and Hybrid A1 shows the necessity of clarifying the common and specific attributes of different tasks. Sharing only the common attributes of tasks in the feature-extraction process leads to a better performance in-depth estimation. We also verify the standard deviation of the performance of these methods among all testing samples to ensure the statistical significance of the results. Since very similar results are observed, we do not present them in Table 4 for conciseness.

### 5.2. Indoor Scene

Road scene images have relatively limited variation in terms of the involved semantics and their spatial arrangements. They are usually captured by a camera fixed on a moving vehicle where the view direction of the camera is always parallel to the ground. This limits the variability of road scene images and makes it easier for the convolutional networks to learn to segment them robustly. In comparison, images of indoor scenes are more complex due to the free view point, the larger number of semantics in the scene, widely varying sizes of objects and their various spatial arrangements. On the other hand, although indoor scenes have smaller depth range than road scenes, they usually have more complex spatial layout, which provides challenges for depth estimation.

In this section, we evaluate the proposed architectures on indoor scene data for both semantic segmentation and depth estimation. We employ RGB-D Scene Understanding Benchmark dataset [34] (SUN-RGBD) for the experiments. SUN-RGBD contains over 10k RGB-D images of indoor scenes captured by 4 types of depth sensors, including also RGB-D images from NYU depth v2 [35], Berkeley B3DO [36], and SUN3D [37]. It provides 2D ground truth object labels for 37 indoor scene classes, such as wall, floor, ceiling, table, chair etc. and depth maps of different resolutions. Our task is to segment the objects within these 37 classes in each image while estimating its depth. In practice, we split the dataset into 5285 training and 5050 testing images, following the experiment configuration introduced in [13].

Similarly to the experiments in Cityscapes dataset, we perform training data augmentation by random cropping, flipping, and mirroring the original training images. However, in the testing phase, instead of cropping the test image as we did in the Cityscapes dataset, we downsample the test image to fit the input size of the hybrid architecture. Since the difference between the size of the test image and input size is not large in SUNR-GBD dataset, directly downsampling the test image to fit the input size strongly improves the efficiency in the testing phase, while not losing the important information in the test data.

#### 5.2.1. Semantic Segmentation

**Figure 7:**
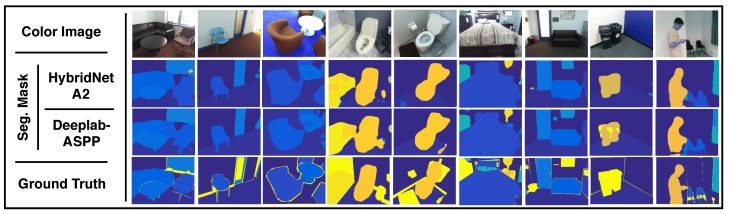
Semantic segmentation qualitative results. A comparison between semantic segmentation estimations against ground truth is presented. Input image is depicted in the first row. In the 2nd and 3rd row, the estimated segmentation mask obtained from HybridNet A2 and the ground truth are presented, respectively.

SUN-RGBD is a very challenging indoor scene dataset for semantic segmentation, in which object classes come in various shapes, sizes, and different poses. There are also frequent partial occlusions between objects, which is typical in indoor scenes, due to the fact that many object classes are presented in each of the test images. Figure 7 provides a visual comparison for the estimated segmentation mask against ground truth. The figure presents, row-wise, 7 out-of-training examples where the first row shows the input images, the 2nd and 3rd row show the estimated segmentation mask from HybridNet A2 and DeepLab-ASPP respectively, and the last row shows the ground truth. HybridNet A2 exhibits stronger performance in distinguishing different objects in indoor scenes compared to DeepLab-ASPP.

Additionally, to qualitative results, we follow the three metrics introduced in Section 5.1.1: the global accuracy (G), the class average accuracy (C) and mean intersection over union (mIoU) to evaluate the segmentation performance quantitatively. We also benchmark the proposed architectures against several other well adopted architectures for semantic segmentation, such as FCN [11], SegNet [13], DeepLab [16] and DeconvNet [38]. For FCN, the parameters for the deconvolutional layers are learned from the training process instead of using fixed parameters to perform bilinear upsampling. For DeepLab, three architectures are employed, which are DeepLab-ASPP, DeepLab-LargeFOV, and DeepLab-LargeFOV-denseCRF. They use the same VGGNet architecture for feature map extraction, which is similar to the proposed architectures. DeepLab-LargeFOV performs single scale upsampling on the feature map, while DeepLab-ASPP performs multi-scale upsampling. DeepLab-LargeFOV-denseCRF introduces a dense conditional random field as a post-processing step for DeepLab-LargeFOV. Table 5 shows the quantitative results of the proposed architectures (HybridNet A1 and A2) compared with other methods. HybridNet A2 achieves the best results in C and mIoU over all the 7 methods while also obtaining a (71.63%) in G close to the best (73.87%) obtained in DeepLab-ASPP. The higher global accuracy and lower per-class accuracy obtained in DeepLab-ASPP in comparison to HybridNet A2 illustrates that DeepLab-ASPP prefers to better cover large objects in the scene such as floor and wall, which provides good results in global evaluation. However, this affects its performance in smaller objects, which results in its lower per-class accuracy, as well as mIoU. The improvement against DeepLab-ASPP verifies again the idea of the multi-task learning, that estimating depth in addition to semantic segmentation helps the segmentation task (6.1% and 5.1% improvement in C and mIoU respectively). The performance of HybridNet A1 is even worse than the single-task method DeepLab-ASPP, which indicates that the idea of benefiting from unifying two single tasks in a hybrid architecture can hardly be achieved by simply sharing the feature-extraction process in more complex indoor scenes. The best segmentation performance obtained by HybridNet A2 compared with HybridNet A1 shows the importance of selecting a suitable unifying strategy in a multi-task learning problem and verifies the efficiency of the strategy employed in HybridNet A2.

#### 5.2.2. Depth Estimation

**Figure 8:**
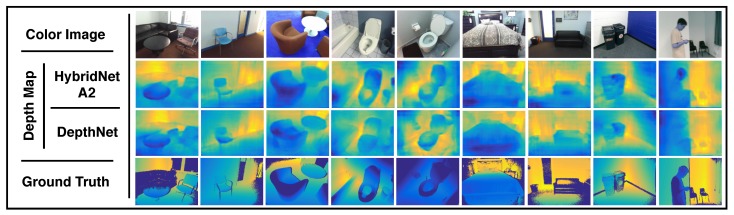
Depth estimation qualitative results. A comparison between depth estimations against ground truth is presented. Input image is depicted in the first row. The 2nd, 3rd and 4th rows present the estimated depth map of our method, DepthNet and the ground truth, respectively.

For depth estimation evaluation Figure 8 depicts a qualitative analysis of results. The figure presents, column-wise, the same 7 out-of-training examples presented in Figure 7, where the first row shows the input images, the 2nd and 3rd row show the estimated depth map from HybridNet A2 and DeepLab-ASPP respectively, and the last row shows the ground truth. The depth maps estimated by HybridNet A2 are more consistent with the ground truth than those obtained by DepthNet in terms of the depth layering.

Additionally, to qualitative analysis, we evaluate the performance following the metrics introduced in Section 5.1.2: PP, Mean Variance Normalized Pixel of Percentage (PP-MVN), ARD, SRD, Linear Root Mean Square Error (RMSE-linear), Log Root Mean Square Error (RMSE-log) and SIE. Table 6 shows the quantitative results of the proposed architectures (HybridNet A1 and A2) and DepthNet under different metrics. HybridNet A2 outperforms over all the metrics which proves that performing semantic segmentation in addition to depth estimation helps the depth estimation task. The better performance of HybridNet A2 in comparison to A1 confirms the efficiency of the unifying strategy proposed in HybridNet A2 in more complex indoor scenes.

#### 5.2.3. Comparison with Other Hybrid Architectures

**Figure 9:**
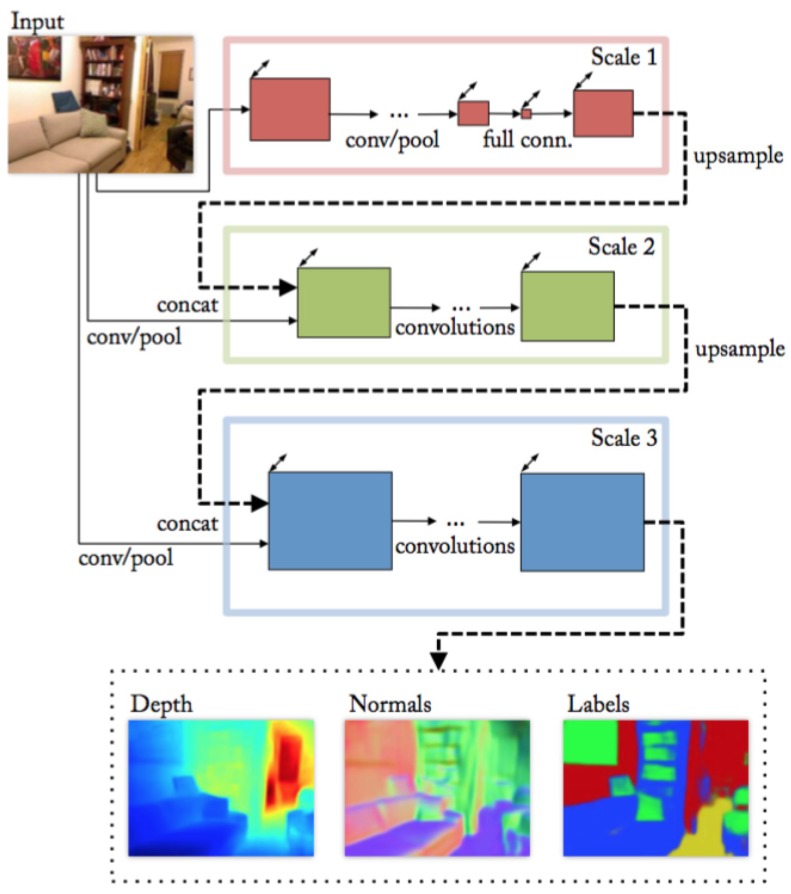
The hybrid architecture proposed in Eigen [21].

To compare HybridNet A2 with other hybrid architectures in the state of the art, the method proposed in [21] is chosen. This method addresses three different tasks including semantic segmentation, depth estimation, and surface normal estimation. The architecture is designed as a stacking of three VGG structures [28] representing different scales of feature extraction (shown in Figure 9). Each of the VGG structures takes the output of the previous one along with the input color image as its input. Among the three tasks, depth estimation, and surface normal estimation are two tasks tackled jointly, which means that these two tasks share the network in scale 1 while the networks in scale 2-3 are separately assembled for each task. For the semantic segmentation task, the architecture shown in Figure 9 is used again. However, different from the other two tasks, the architecture of semantic segmentation allows two additional input channels which are depth and normal channels. This architecture is only fine-tuned from the model previously trained on depth and normal estimation to generate semantic segmentation masks.

Although the source code of this method was not available, the performance evaluation is reported in a public dataset (NYU Depth V2 dataset [35]). To make the comparison with this approach, we trained and evaluated our approach on NYU Depth V2 dataset. This data set includes RGB images and their corresponding 2D ground truth object labels for 40 indoor scene classes and depth map. NYU depth V2 dataset is divided into 795 images for training and 654 for testing. Due to the small number of images available for training, we augment the training set by random cropping, flipping, and mirroring.

Table 7 and Table 8 show the quantitative results of HybridNet A2 for both tasks and provides a comparison with the approach proposed in [21], denoted as Eigen. Semantic segmentation results in Table 7 show that HybridNet A2 outperforms Eigen in class average accuracy (C) and mean intersection over union (mIoU) while keeping similar results than Eigen in Global accuracy (G). It also illustrates that addressing RGB-D-based semantic segmentation task under a multi-task learning scheme better uses the depth information than directly feeding the depth information to the network as an extra input channel. On the other hand, depth estimation results in Table 8 show that HybridNet A2 has a better performance in the relative measure SIE, while in the absolute measures Eigen outperforms HybridNet A2. The better performance of HybridNet A2 in the relative measure shows that HybridNet A2 has a better depth layering capability than Eigen, which is more relevant in real applications. For absolute measures, we believe that the worse performance of HybridNet A2 is due to the weaker ability in describing the global layout of the scene. HybridNet A2 employs a much simpler architecture (AlexNet structure) for global depth network compared with the network of scale 1 (VGG structure) in Eigen.

## 6. Conclusions and Future Work

In this paper, we have introduced a methodology for depth estimation and semantic segmentation from a single image using a unified convolutional network. The main goal of the proposed method is to seek for a better hybrid architecture of CNNs that modularizes the feature-extraction process by separating it into distinct feature extraction for a specific task and common feature extraction for both tasks. In this manner, both tasks can benefit from the extracted common features without being affected by those features only relevant to one task, which leads to a better performance. We also prove that solving correlated tasks such as semantic segmentation and depth estimation together can improve the performance of methods tackling the tasks separately.

The qualitative and quantitative results shown in Section 5 demonstrate that the unifying strategy employed in HybridNet A2 produces a better hybrid architecture for semantic segmentation and depth estimation compared to Hybrid A1. Hybrid A2 outperforms the results obtained by single-task approaches, which proves that sharing underlying feature extraction helps to improve the final performance in both tasks. Likewise, it is also proved that our methodology obtains comparable results to benchmarking hybrid approaches.

On the other hand, there are also some interesting problems pending for a future study:

- *Designing better loss functions for a multi-task learning scheme.* The loss function employed in the state-of-the-art approaches is normally a balanced linear combination of losses for single tasks. However, these losses may have totally different physical meaning regarding the tasks (e.g., cross entropy and Euclidean loss), which makes it hard to combine them. Finding higher level evaluation metrics helps define the loss function for a multi-task learning system. For instance, evaluating on the prediction of the 3D oriented bounding box of objects requires using both semantic segmentation and depth estimation result, which naturally combines the loss function for both tasks.
- *Applying to higher level tasks requiring 3D analysis.* Since the proposed approach produces an object level segmentation and a depth map of an input image, applying the estimated result to applications requiring 3D analysis (such as traffic violation detection) will be of great interest.

## Figures and Tables

**Table 1:** A comparison of different types approaches.

	Unsupervised	Object Level Segm.	Depth Estimation	Joint Estimation
Ours	×	✓	✓	✓
Eigen [21]	×	✓	✓	×
PLEDL [25]	×	✓	✓	✓
Superpixel [4,5]	✓	×	×	-
Active Contour [6,7]	✓	×	×	-
Watershed [8,9]	✓	×	×	-
Semantic Segm. [10,11,13,16,26]	×	✓	×	-
Depth Prediction [19]	×	×	✓	-

**Table 2:** Evaluation of HybridNet against Multi-task and single-task approaches (best results in bold).

	G	C	mIoU
HybridNet A2	**93.26**	**79.47**	**66.61**
HybridNet A1	89.31	77.22	58.1
PLEDL [25]	-	-	64.3
DeepLab-ASPP [16]	90.99	74.88	58.02
FCN [11]	-	-	65.3
SegNet [13]	-	-	57.0
GoogLeNetFCN [26]	-	-	63.0

**Table 3:** Definition of the evaluation metrics: for depth estimation: Percentage of Pixel (PP), PP-MVN Absolute Relative Difference (ARD), Square Relative Difference (SRD), RMSE-linear, RMSE-log and Scale-Invariant Error (SIE).

Metrics	Definition
PP	$max\left(\frac{d_{i}}{d_{i}^{*}},\frac{d_{i}^{*}}{d_{i}}\right)=\gamma <threshold$
PP-MVN	$max\left(\frac{MVN\left(d_{i}\right)}{MVN\left(d_{i}^{*}\right)},\frac{MVN\left(d_{i}^{*}\right)}{MVN\left(d_{i}\right)}\right)=\gamma <threshold$
ARD	$\frac{1}{N}\sum \left|d_{i}-d_{i}^{*}\right|/d_{i}^{*}$
SRD	$\frac{1}{N}\sum {\left|d_{i}-d_{i}^{*}\right|}^{2}/d_{i}^{*}$
RMSE-linear	$\sqrt{\frac{1}{N}\sum {∥d_{i}-d_{i}^{*}∥}^{2}}$
RMSE-log	$\sqrt{\frac{1}{N}\sum {∥log\left(d_{i}\right)-log\left(d_{i}^{*}\right)∥}^{2}}$
SIE	$\frac{1}{N}\sum_{i}{\left(log\left(d_{i}\right)-log\left(d_{i}^{*}\right)+\frac{1}{N}\sum_{j}\left(log\left(d_{j}\right)-log\left(d_{j}^{*}\right)\right)\right)}^{2}$

**Table 4:** Depth estimation. Quantitative evaluation: PP, PP-MVN, ARD, SRD, RMSE-linear, RMSE-log, and SIE (best results in bold).

	HybridNet A2	HybridNet A1	DepthNet [19]	
γ<1.25 (MVN)	**0.7483**	0.6834	0.7248	higher is better
γ<1.25	0.5968	0.5037	**0.6048**	
$\gamma <1.25^{2}$	**0.8221**	0.8172	0.8187	
$\gamma <1.25^{3}$	**0.9292**	0.9194	0.9152	
ARD	0.24	0.2879	**0.23**	lower is better
SRD	**4.27**	4.35	4.43	
RMSE-linear	**12.09**	12.67	12.35	
RMSE-log	0.4343	**0.3407**	0.4340	
SIE	**0.19**	0.2	0.25	

**Table 5:** Semantic segmentation. Quantitative evaluation (best results in bold).

	G	C	mIoU
HybridNet A2	71.63	**46.20**	**34.30**
HybridNet A1	69.34	38.64	28.68
DeepLab-ASPP [16]	**73.87**	40.09	29.22
SegNet [13]	72.63	44.76	31.84
DeepLab-LargeFOV [16]	71.90	42.21	32.08
DeepLab-LargeFOV-denseCRF [16]	66.96	33.06	24.13
FCN(learned deconv) [11]	68.18	38.41	27.39
DeconvNet [38]	66.13	32.28	22.57

**Table 6:** Depth estimation. Quantitative evaluation: PP, PP-MVN, ARD, SRD, RMSE-linear, RMSE-log, and SIE (best results in bold).

	HybridNet A2	HybridNet A1	DepthNet	
γ<1.25 (MVN)	**89.63**	62.81	83.59	higher is better
γ<1.25	**61.33**	38.63	57.73	
$\gamma <1.25^{2}$	**89.17**	69.38	87.42	
$\gamma <1.25^{3}$	**97.43**	86.28	97.08	
ARD	**0.202**	0.301	0.218	lower is better
SRD	**0.186**	3.02	0.204	
RMSE-linear	**0.682**	8.35	0.715	
RMSE-log	**0.25**	0.432	0.27	
SIE	**0.122**	0.316	0.126	

**Table 7:** Quantitative segmentation results on NYU V2: G, C and mIoU (best results in bold).

	G	C	mIoU
HybridNet A2	64.7	**48.4**	**36.5**
Eigen [21]	**65.6**	45.1	34.1

**Table 8:** Depth estimation results on NYU V2 Quantitative evaluation: PP, PP-MVN, ARD, SRD, RMSE-linear, RMSE-log, and SIE (best results in bold).

	HybridNet A2	Eigen [21]
γ<1.25 (MVN)	0.7293	-
SIE	**0.1571**	0.171
γ<1.25	0.5006	**0.769**
$\gamma <1.25^{2}$	0.8013	**0.95**
$\gamma <1.25^{3}$	0.9422	**0.98**
ARD	0.2787	**0.158**
SRD	0.3236	**0.121**
RMSE-linear	0.9423	**0.64**
RMSE-log	0.3219	**0.214**
